# Adherence to Antihypertensive Therapy and Its Determinants: A Systematic Review

**DOI:** 10.7759/cureus.59532

**Published:** 2024-05-02

**Authors:** Pedro D Ferreira, Jose A Simoes, Denise C Velho

**Affiliations:** 1 Family Medicine, ULS Santo António, USF Santa Maria, Porto, PRT; 2 Department of Medical Sciences, Faculty of Health Sciences, University of Beira Interior, Covilhã, PRT; 3 Family Medicine, ULS de Leiria, USF Santiago, Leiria, PRT

**Keywords:** telemedical technology, mobile apps (mhealth), preventive health, antihypertensive agents, primary health care, medication adherence strategies, antihypertensive therapy, hypertension

## Abstract

Hypertension is a globally prevalent condition, and low adherence to antihypertensive therapy is considered one of the main causes of poor blood pressure (BP) control. Non-adherence to antihypertensive treatment is a complex issue that can arise from various factors; however, gaining an understanding of this provides key targets for intervention strategies. This study aimed to provide an overview of the current status and recent developments regarding our understanding of the determinants of patients' adherence to antihypertensives. A systematic review was performed using the electronic databases MEDLINE/PubMed, Web of Science, Scientific Electronic Library Online (SciELO), and “Índex das Revistas Médicas Portuguesas”, which included studies published between 2017 and 2021 following the PICOS model: (P) Adult patients with the diagnosis of primary hypertension, using at least one antihypertensive agent; (I) all interventions on both pharmacological and non-pharmacological level; (C) patient’s adherence against their non-adherence; (O) changes in adherence to the therapeutic plan; and (S) any study design (except review articles) written in English, French, Spanish or Portuguese.

Articles were reviewed by two researchers and their quality was assessed. Subsequently, determinants were classified according to their consistent or inconsistent association with adherence or non-adherence. Only 45 of the 635 reports identified met the inclusion criteria. Adherence was consistently associated with patient satisfaction with communication, patient-provider relationship, their treatment, and use of eHealth and mHealth strategies; a patient’s mental and physical health, including depression, cognitive impairment, frailty, and disability, previous hospitalization, occurrence of vital events; drug treatment type and appearance; and unwillingness due to health literacy, self-efficacy, and both implicit and explicit attitudes towards treatment. There were discrepancies regarding the association of other factors to adherence, but these inconsistent factors should also be taken into account. In conclusion, the barriers to adherence are varied and often interconnected between socioeconomic, patient, therapy, condition, and healthcare system levels. Healthcare teams should invest in studying patients’ non-adherence motives and tailoring interventions to individual levels, by using a multifaceted approach to assess adherence. Further research is needed to analyze the impact of implicit attitudes, the use of new technological approaches, and the influence of factors that are inconsistently associated with non-adherence, to understand their potential in implementing adherence strategies.

## Introduction and background

Cardiovascular diseases (CVD) are considered a global public health problem and remain the leading cause of death and incapacity worldwide; they account for 32% of all global deaths, of which 85% are attributed to stroke and heart attacks [1]. In 2019, across OECD (Organization for Economic Co-operation and Development) countries, CVD emerged as the number one treatable cause of mortality, representing 36% of premature deaths amenable to treatment, and the third disease with most preventable deaths through effective public health measures and primary care intervention (19% of all preventable deaths) [2]. Despite the significant improvements in CVD mortality rates, evidence from some countries has shown a slowdown in the reduction of this rate, even before the coronavirus disease 2019 (COVID-19) pandemic, due to certain challenges including the rapid ageing of the population and the difficulty in promoting practices to deal with some risk factors, such as obesity and diabetes [3].

In line with worldwide trends, CVD represents the main cause of mortality in Portugal (29.9% of all deaths in 2019) [4,5], with arterial hypertension emerging as the most important risk factor for several complications such as ischaemic heart disease, stroke, chronic kidney disease and dementia [6-8]. Additionally, in light of the current epidemiological scenario we are living in, it is important to note that high blood pressure (HBP) increases the risk of severe COVID-19 infection [9,10]. In 2014, 25.3% (2.2 million people) reported having HBP in a Portuguese national survey, with the majority of them being females [11].

The diagnosis of hypertension is established based on the following parameters: systolic blood pressure (SBP) ≥140 mmHg and/or diastolic blood pressure (DBP) ≥90 mmHg [12]. Achieving good control of blood pressure (BP) is possible by using both non-pharmacological and pharmacological treatment, and studies have proven the efficacy of these interventions [13]. The impact of lowering BP can be so relevant that even modest improvements in SBP (reduction of 10 mmHg) or DBP (reduction of 5 mmHg) can have an appreciable effect on health outcomes, reducing the risk of cardiovascular events [14]. The approach to good BP control depends on multiple factors, including pathophysiological and pharmacological aspects, levels of adherence, and therapeutic inertia [15]. However, despite substantial efforts in promoting healthy lifestyles and the major progress made in pharmacological treatment, low adherence to preventive measures and prescribed medication is considered one of the main causes of the lack of BP control in many individuals [16]. A systematic review and meta-analyses published in 2017 estimated that 45.2% of hypertensive patients fail to adhere to prescribed regimens, especially among patients with uncontrolled BP (83.7%), highlighting the importance of addressing low adherence to the therapeutic plan in BP management [17].

The World Health Organization defines adherence to long-term therapy as “the extent to which a person’s behaviour - taking medication, following a diet, and/or executing lifestyle changes, corresponds with agreed recommendations from a healthcare provider”. The reported rates of adherence to pharmacotherapy oscillate between 50 and 70%, depending on the group being studied, the follow-up duration, the methods applied for adherence evaluation, and the different drug classes involved [18]. In the process of adherence, both patient and provider understand and agree with the recommendations. However, it is not unusual that this term is used as a synonym for compliance. This last concept brings a different connotation since it implies passive behaviour from the patient who is willing to follow the provider’s recommendations, despite not necessarily agreeing with them [19].

The complexity surrounding a patient’s behaviour towards the prescribed therapy cannot easily be captured in a single word, as it tends to amplify the clinician’s control over the process of taking medications and underrate the patient’s beliefs, personal circumstances, and available resources [20]. Adherence often emerges as a multidimensional phenomenon, which goes beyond the misleading idea that only the patient is responsible for taking their treatment, and can be classified into five dimensions that might interact with each other: patient-related factors, condition-related factors, therapy-related factors, social/economic-related factors, and health system/healthcare team-related factors [18]. The acknowledgement of the complexity regarding the determinants of patients’ adherence allows us to avoid exclusively blaming the patients for their non-adherence and guide health systems and healthcare teams to identify effective solutions, which is critical in reducing the burden related to hypertension and its complications.

Given the importance of medication adherence (MA) in the control of BP and the critical role of different factors in patient adherence, this study aims to provide an overview, via a systematic approach, of the current status and recent developments regarding the determinants of patient adherence to treatment in the adult population, by assessing and comparing different predictors of adherence to both pharmacological and non-pharmacological treatment for hypertension.

## Review

Materials and methods

Design and Registration

We conducted a systematic review of all scientific articles available (according to the search strategy defined below) and followed the Cochrane Handbook, a methodology guide for systematic reviews. The protocol has been registered in the International Prospective Register of Systematic Reviews (PROSPERO) under the number CRD42022301595, and the study was conducted based on the Preferred Reporting Items for Systematic Reviews and Meta-Analyses (PRISMA) guidelines.

Search Strategy

A search for relevant articles published between January 1, 2017, and December 31, 2021, was performed on electronic databases such as Medline (via PubMed), Web of Science, Scientific Electronic Library Online (SciELO), and other national/regional databases (Índex - Revistas Médicas Portuguesas).

The general search terms used on Pubmed were ((("Patient Compliance"[Title/Abstract] OR "Medication Adherence"[Title/Abstract]) OR ("Patient Compliance"[Majr] OR "Medication Adherence"[Majr])) AND ((high blood pressure [Title/Abstract] OR hypertension [Title/Abstract]) OR ("Hypertension"[Majr]))) AND ("Antihypertensive Agents"[Majr]). The search was restricted to articles written in English, French, Spanish, and Portuguese, whose abstracts were available online and involved the adult population. A similar search strategy was used in other databases, but the MeSH terms and text words were modified/adapted as per the specific requirements of each database.

The research question was elaborated using the PICOS model: (P) Adult patients with the diagnosis of primary hypertension, using at least one antihypertensive agent; (I) all interventions on both pharmacological and non-pharmacological levels; (C) patient’s adherence against their non-adherence; (O) changes in adherence to the therapeutic plan; and (S) any study design (except review articles) written in English, French, Spanish or Portuguese: “What influences patient’s adherence to antihypertensive therapies?” The reviewing team’s decision to include adherence to both pharmacological and non-pharmacological treatment was based on the fact that the approach in previous studies frequently focuses on determinants of both types of treatment. Thus, the conclusions would be more reliable since treatment adherence carries these two dimensions together.

Eligibility Criteria

Studies meeting the following criteria were included: (i) type of study: any study design conducted in English, French, Spanish or Portuguese, preferring those with a higher level of evidence [i.e., randomized controlled trials (RCT)], except for all types of reviews; (ii) type of population: adult patients aged 18 years or over, of any gender or ethnicity, with a diagnosis of primary hypertension, as well as participants with other comorbidities (e.g., diabetes); (iii) type of intervention: adherence to both pharmacological and non-pharmacological treatment using direct or indirect measures of adherence and/or based on patient’s perspective of the impact on their quality of life, changes in the pharmacological treatment, combination of both pharmacological and non-pharmacological treatment, adherence aids (e.g., reminders/alarms), and use of educational measures (e.g., communication between the patient and the healthcare professional); articles examining adherence to multiple pharmacological therapies were deemed eligible for inclusion if antihypertensive therapy was among the different pharmacotherapeutic agents being studied; (iv) type of comparator: patient’s adherence against the non-adherence; (v) type of outcome: primary outcome: the adherence to the antihypertensive therapy measured using the different tools available; secondar outcomes: the reduction and/or control of the systolic/diastolic blood pressure and patient-reported information on quality of life or symptoms.

The exclusion criteria were as follows: (i) studies in languages other than the ones in the inclusion criteria, duplicate articles from different databases, and articles whose titles and/or abstracts did not meet the inclusion criteria. Articles that required payment for access were analyzed on a case-by-case basis and excluded if they were not deemed exceptionally relevant to this study. Review articles were excluded, to avoid a high risk of duplicating the results; (ii) studies involving patients under 18 years of age, patients with secondary hypertension (e.g., pre-eclampsia, hyperaldosteronism), and pregnant or breast-feeding women; (iii) studies involving other interventions besides pharmacological and non-pharmacological treatments (i.e., surgical).

Study Selection

All of the retrieved articles were organized and screened using the online software Covidence and the Endnote Web reference management software. After removing duplicates, the articles’ titles and abstracts were screened by two independent reviewers who were blinded to each other’s decisions. The articles and abstracts that matched the objective of this review were eligible for a full-text review. The two authors assessed the eligibility criteria and applied them to the final articles and those that met the inclusion criteria were retrieved. Finally, studies that did not meet any of the inclusion criteria were excluded. Disagreements were always solved by reaching a consensus and, therefore, there was no need to involve a third author in the selection process. Finally, after browsing the reference lists, the data were extracted from each study to synthesize their findings.

Data Extraction and Management

Data extraction was performed by one investigator and included the following items: authors, date of the study, study design, research aim, sample size, mean age, tool used in the measurement of adherence, and definition of adherence. To summarize the major findings and the direction of the associations with MA, data regarding the different determinants affecting adherence were organized into five domains: patient characteristics, socioeconomic status, comorbidities-related, therapy-related, and healthcare/health system issues. For each domain, subcategories were introduced according to the determinants present in the included studies. In addition, to systematize the information, factors were organized into three columns to signify whether they had a statistically significant association with adherence, a statistically significant association with non-adherence, or a non-significant association at all.

Quality of Evidence and Risk of Bias

To ensure the quality of the analyzed articles, all eligible studies were reviewed by two researchers to appraise their risk of bias, using different tools according to the study design of the article being evaluated. For RCTs, we applied the “Rob2: Cochrane risk-of-bias tool” that assessed the following five components: (i) randomization process, (ii) deviations from the intended interventions, (iii) missing outcome data, (iv) measurement of the outcome, and (v) selection of the reported results. The quality assessment for each component was classified into three possible categories (high risk of bias, some concerns, and low risk of bias) and an overall judgment was stated. For cohort studies, we applied the “ROBINS-I: Cochrane risk-of-bias tool for non-randomized studies of interventions”, which has seven components: (i) confounding, (ii) selection bias, (iii) classification of intervention, (iv) intended intervention, (v) missing data, (vi) measures of outcome, and (vii) reported results. The evaluation was expressed in five possible outcomes (low risk, moderate risk, serious risk, critical risk, and no information). Finally, cross-sectional studies were evaluated using the “JBI Critical Appraisal Checklist for Analytical cross-sectional studies”, which involved eight questions with the following possible answers: “Yes”, “No” and “Unclear”. The assessment of the risk of bias was based on the percentage of “yes” scores, considering the following three levels of classification: high quality (>70% of “yes” scores), moderate quality (from 50 to 69%), and low quality (<50%).

Results

Study Selection

**Figure 1:**
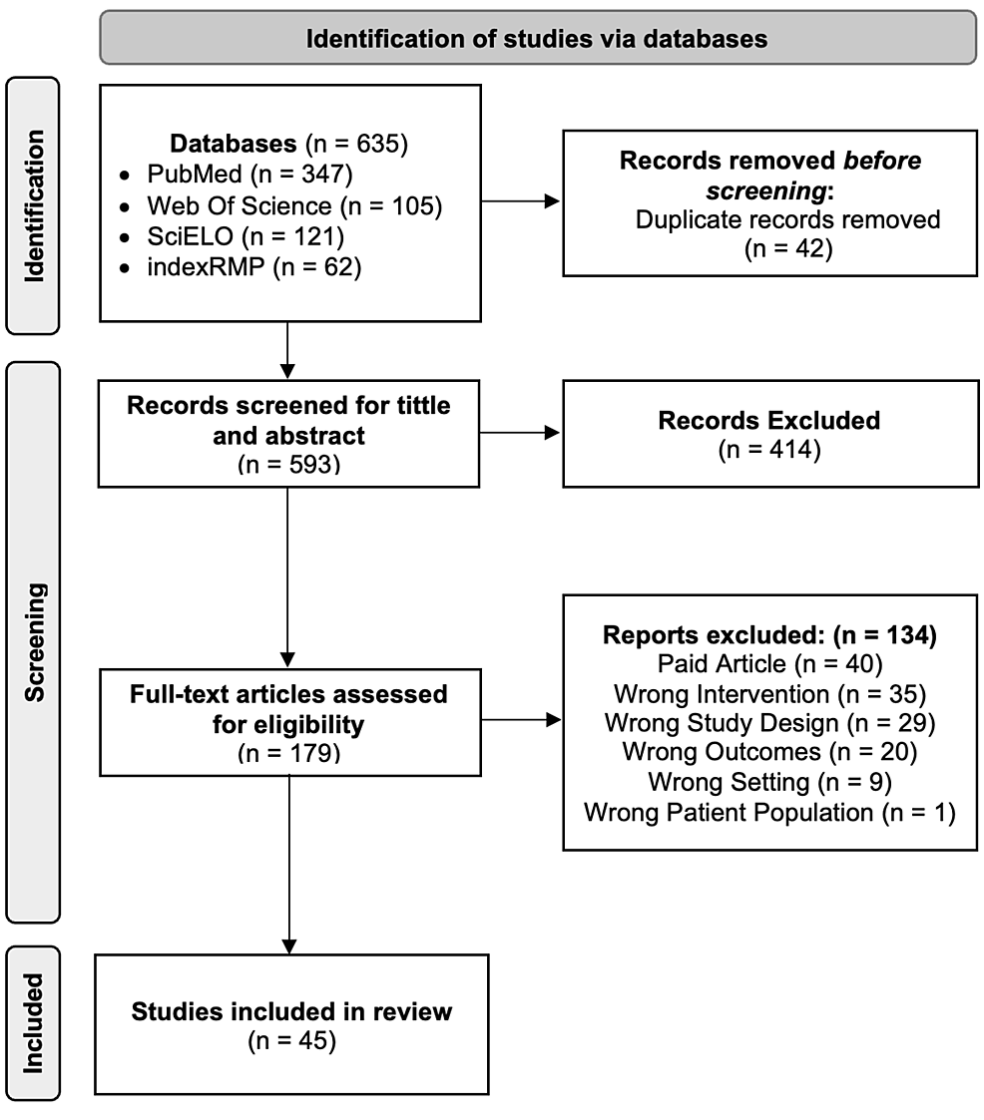
PRISMA flow chart depicting the literature search and study selection PRISMA: Preferred Reporting Items for Systematic Reviews and Meta-Analyses

The literature search on the reported databases elicited 635 articles. After removing 42 duplicates, 593 articles qualified for a title and abstract screening, and according to the inclusion criteria, 179 underwent full-text review. A detailed assessment of the 179 full-text articles resulted in the elimination of an additional 134. Finally, 45 articles that fully met the eligibility were included in this review. Figure 1 shows the PRISMA flow chart illustrating the search and study selection.

Study Characteristics

The characteristics of the included studies on adherence to antihypertensive therapy are presented in Table 1 [21-65]. Overall, we included 21 cross-sectional studies [22-24,28,30-32,35,36,40,41,45,47,48,50-52,56,57,62,64], 19 cohort studies [21,26,27,29,33,34,37-39,42-44,49,53-55,58,60,65], four RCTs [25,46,59,61], and one quasi-experimental study [63]. The total number of patients included for analysis was 1,533,220, with individual study sample sizes ranging from 32 to 484,493 participants. The mean age of the participants ranged from 46.5 to 76.6 years old, with most of the studies having a population with a mean age above 50 years.

**Table 1:** Characteristics of included studies on adherence to antihypertensive therapy CMA: cumulative medication adherence; K-Wood-MAS-4: Krousel-Wood Medication Adherence Scale; MA: medication adherence); MARS: medication adherence rate scale; MGL: Morisky Green Levine; MMAS-8: Morisky Medication Adherence Scale with 8 items; MPR: medication possession ratio; MRA: medication refill adherence; PDC: proportion of days covered; PDT: percentage of doses taken; PDTc: percentage of doses taken correctly; PMC: proportion of months covered; TDM: therapeutic drug monitoring

Author	Year	Study design	Study objective	Sample size (N); mean age (A)	Measurement of adherence	Definition of adherence
Arancón-Monge et al. [21]	2020	Cohort study	To study if changes in appearance and name in bioequivalent drugs are associated with non-adherence to antihypertensive and lipid-lowering medications	N = 274 patients; A = 72 years	MGL (scores ranged from 0 to 4); direct count of the medication	MGL: high adherence (score of 4). Direct count: non-adherence if the patient forgets to take more than 2 doses/trimester
Avataneo et al. [22]	2018	Cross-sectional study	To assess adherence to treatment in patients with reported resistant hypertension using therapeutic drug monitoring and to study parameters that might predict non-adherence	N = 50 patients; A = 56 years	TDM specialist opinion. A homemade questionnaire with 10 items (scores ranged from 0 to 10)	TDM: fully adherent patients – detectable plasma concentrations of all prescribed drugs. Specialist opinion: comment on patient adherence based on their personal experience before knowing TDM results. Questionnaire: high adherence (scores ranged from 9 to 10)
Barbosa et al. [23]	2019	Cross-sectional study	To evaluate predictors of adherence to antihypertensive therapies in adults/elderly	N = 257 patients; A = Not reported	A questionnaire with 24 items, that accessed four dimensions: patient, disease/treatment, healthcare service, and environment (scores ranged from 24 to 120)	Adherent (score of 73–120)
Berhe et al. [24]	2017	Cross-sectional study	To evaluate the impact of adverse drug events and treatment satisfaction on antihypertensive medication adherence	N = 925 patients; A = 57 years	MMAS-8 (scores ranged from 0 to 8)	High adherence (score of 8)
Chandler et al. [25]	2019	Randomized controlled trial	To evaluate the efficacy of the SMASH (smartphone app) in establishing systolic blood pressure control via increased medication adherence	N = 54 patients; A = 46.5 ± 9.9 years	MMAS-8 (scores ranged from 0 to 8); medication tray: timestamps of openings of pill tray compartments (scores ranged from 0 to 1)	MMAS-8: high adherence (score of 8). Medication tray: adherent if doses are taken within 0-3 hours (score of 1)
Chang et al. [26]	2021	Cohort study	To study the association between the patient-clinician relationship and adherence to antihypertensive medications among black adults with hypertension	N = 2571 patients; A = 58 years	MRA	Adherent if MRA ≥80%
Cho et al. [27]	2018	Cohort study	To study the association between cognitive function and antihypertensive medication adherence in elderly patients without dementia	N = 42,132 patients; A = not reported	CMA using prescription data	Adherent if CMA ≥80%
Craig et al. [28]	2021	Cross-sectional study	To study the contributions of implicit and explicit attitudes in explaining differences between both objective and subjective antihypertensive medication adherence measures	N = 85 patients; A = 62.3 years	PDC K-Wood-MAS-4 (scores ranged from 0 to 4)	Non-adherent if PDC <0.8 or K-Wood-MAS-4 ≥1
Del Pinto et al. [29]	2021	Cohort study	To assess antihypertensive treatment adherence and BP control rates in treated hypertensive elderly patients according to different treatment patterns	N = 13,196 patients; A = 73.2 ± 7.5 years	The ratio between the number of pills in boxes used and those expected in 6 months	Adherent if rate ≥80%
Durand et al. [30]	2018	Cross-sectional study	To study predictors of long-term medication adherence in patients who appear to have treatment-resistant hypertension using a composite adherence score	N = 204 patients; A = 69.9 years	Adherence composite of MMAS-8 (scores ranged from 0 to 8) and MARS (scores ranged from 0 to 10); prescription refill records; biochemical assay of urine (TDM)	Non-adherent if: MMAS-8 <6 and/or MARS <23. Prescription refill records <80%. Urine sample: undetectable concentrations of all the prescribed drugs
Fang et al. [31]	2020	Cross-sectional study	To study the association of cost-related medication non-adherence barriers and hypertension management among US adults with self-reported hypertension	N = 7498 patients; A = not reported	A questionnaire with 3 items to assess cost-related medication non-adherence	Non-adherent if answered “yes” to any of the 3 questions
Fernandez et al. [32]	2017	Cross-sectional study	To study factors related to non-adherence to pharmacological treatment and to design an educational program	N = 102 patients; A = not reported	MGL (scores ranged from 0 to 4)	High adherence (score of 4)
Fortuna et al. [33]	2018	Cohort study	To study the association between the patient experience with care and medication adherence	N = 2128 patients; A = 50.4 years	MMAS-8 (scores ranged from 0 to 8)	High adherence (score of 8)
Gao et al. [34]	2020	Cohort study	To study the determinants of antihypertensive medication adherence	N = 7638 patients; A = 67.6 ± 11.1 years	PDC	Good adherence if PDC >0.8
Gewehr et al. [35]	2018	Cross-sectional study	To study the adherence to antihypertensive agents and the determinants associated with non-adherence in primary healthcare	N = 156 patients; A = 66.9 ± 8.3 years	Brief Medication Questionnaire (scores ranged from 0 to 3)	High adherence (score of 0–1)
Gniwa et al. [36]	2019	Cross-sectional study	To investigate therapeutic adherence and to recognize factors associated with poor adherence	N = 276 patients; A = 64.9 ± 10.2 years	Girerd test (6 items) (scores ranged from 0 to 6)	Good observers (total of yes = 0)
Gupta et al. [37]	2017	Cohort study	To study the therapeutic applications of biochemical screening for the presence of antihypertensive medications in bodily fluids	N = 331 patients; A = 54.3 years (UK patients)/51.4 years (Czech patients)	Blood and urine measure of metabolites using liquid chromatography-tandem mass spectrometry	Adherent patients - detectable concentrations of all prescribed drugs in serum/urine
Gupta et al. [38]	2017	Cohort study	To study the relationships between non-adherence and main demographic and BP-lowering therapy-related factors	N = 1348 patients; A = 55.8 years (UK patients)/54.4 years (Czech patients)	Blood and urine measure of metabolites using liquid chromatography-tandem mass spectrometry	Adherent patients - detectable concentrations of all prescribed drugs in serum/urine
Hargrove et al. [39]	2017	Cohort study	To evaluate whether individual factors predict adherence trajectories and to improve the classification of antihypertensive adherence using group-based trajectory models	N = 282,520 patients; A = 75 years	PMC, PDC	Adherent if PMC ≥80% days and/or PDC ≥80%
Heizomi et al. [40]	2020	Cross-sectional study	To study the characteristics related to health literacy among hypertensive patients, its relationship with adherence to antihypertensive medications and to determine the validity of the four-item MGL scale	N = 300 patients; A = 56.7 years	MGL (scores ranged from 0 to 4)	MGL: high adherence (score of 4)
Ishida et al. [41]	2019	Cohort study	To study treatment patterns and medication adherence to different antihypertensive drug classes and to evaluate whether fixed-dose combinations were being prescribed alone or simultaneously with other antihypertensive drugs	N = 47,891 patients; A = 70.1 years	PDC	High adherence if PDC ≥80%
Kim et al. [42]	2019	Cohort study	To study the effect of single-pill combination on adherence to antihypertensive medication in a real-world setting	N = 116,677 patients; A = not reported	MPR: dividing the total days supplied by the number of days between the first and last refills	No more information was given
Kulkarni et al. [43]	2021	Cohort study	To study pharmacotherapy non-adherence using urine screens among patients with uncontrolled HTN, with analyses of demographics, polypharmacy, medication type, and comorbidities	N = 174 patients; A = 56 years	Urine measure of metabolites using liquid chromatography-tandem mass spectrometry	Adherent patients - detectable concentrations of all prescribed drugs in urine
Lauffenburger et al. [44]	2017	Cohort Study	To evaluate patterns of antihypertensive therapy initiation, compare persistence and long-term adherence to treatments in patients initiating fixed-dose combinations and other antihypertensive therapies, and identify other determinants of adherence and persistence to antihypertensives	N = 484,493 patients; A = 47.2 years	PDC	Fully adherent if PDC >0.8 to at least one antihypertensive
Macquart de Terline et al. [45]	2019	Cross-sectional	To study adherence to medication and association with socioeconomics, clinical and treatment predictors of low adherence among hypertensive patients in 12 sub-Saharan African countries	N = 2198 patients; A = 57.7 ± 12.0 years (women) and 59.2 ± 11.4 years (men)	MMAS-8 (scores ranged from 0 to 8)	High adherence (score of 8)
Morawski et al. [46]	2018	Randomized controlled trial	To study the association between medication adherence and blood pressure control with a smartphone app among patients with uncontrolled hypertension	N = 411 patients; A = 52.0 years	MMAS-8 (scores ranged from 0 to 8)	High adherence (score of 8)
Nascimento et al. [47]	2021	Cross-sectional	To study the factors associated with adherence to the non-pharmacological treatment of hypertension in primary healthcare	N = 421 patients; A = 59.9 ± 11 years	Instrument to evaluate the adherence of participants to non-pharmacological therapies on the following parameters: weight (BMI) and abdominal circumference (AC) control, the practice of physical activity, and alcohol consumption	Patient considered adherent to each measure: weight control: when BMI <25 kg/m^2^ (below 65 years old) or <27 kg/m^2^ (above 65 years old); AC: when AC <80 cm (women) and <94 cm (men); physical activity: who practiced moderate physical activities, aerobic training, or resistance training; alcohol consumption: maximum 2 doses a day (men) and maximum 1 dose (women and low-weight individuals)
Nashilongo et al. [48]	2017	Cross-sectional	To evaluate the levels and predictors of compliance to antihypertensive therapy among patients in primary healthcare and to validate the Hill-Bone compliance scale	N = 120 patients; A = 47.3 ± 11.1 years	Percentage adherence level per patient calculated from the scores on the Hill-Bone Blood Pressure Scale	Perfect adherence (100%) and acceptable adherence (≥80%)
Nishimura et al. [49]	2020	Cohort study	To study the adherence pattern of new users of antihypertensive drugs and to evaluate possible patient and facility characteristics connected with low adherence	N = 31,592 patients; A = 51.7 years	PDC	High adherence if PDC ≥80%
Paczkowska et al. [50]	2021	Cross-sectional	To evaluate the impact of patient knowledge on adherence to medical recommendations/improving the efficacy of hypertension treatment and to analyze sociodemographic/clinical factors that affect the level of patient knowledge regarding hypertension	N = 488 patients; A = 63.7 ± 13	Questionnaire (5 items) regarding treatment adherence	No more information was given
Pan et al. [51]	2019	Cross-sectional	To study the factors influencing adherence behaviors and determine the interventions that improve adherence in hypertensive patients	N = 488 patients; A = not reported	Therapeutic Adherence Scale for Hypertensive Patients (TASHP) with 25 items (scores ranged from 25 to 125)	Low adherence if TASHP <109
Pluta et al. [52]	2020	Cross-sectional	To study the level of acceptance of illness and compliance with therapeutic recommendations in patients with hypertension	N = 200 patients; A = 49.1 ± 11.6 years	MMAS-8 (scores ranged from 0 to 8)	High adherence (score of 8)
Rea et al. [53]	2021	Cohort study	To study the relationship between initial antihypertensive treatment strategy and adherence in patients starting treatment with one drug or a dual single-pill combination	N = 63,448 patients; A = 59 years	PDC	High adherence if PDC >75%
Schoenthaler et al. [54]	2017	Cohort study	To study the effect of patient-provider communication on medication adherence among hypertensive patients in a primary care setting	N = 92 patients; A = 59.7 years	Adaptive statistical modeling (ASM) of electronic monitoring device: division into 5 clusters	Adaptively generated adherence calculated using the ASM methods: very high adherence if fits on cluster 1 (97%) or cluster 2 (93%)
Shani et al. [55]	2019	Cohort study	To study medication adherence to oral antihypertensive medications, to compare adherence rates to different medications, and to study determinants among patients with good adherence	N = 31,530 patients; A = not reported	Rate of pharmacy-filled prescription	Good adherence: purchasing at least 9 monthly prescriptions equals at least 75% adherence
Shi et al. [56]	2019	Cross-sectional	To study the impact of medication literacy among hypertensive patients on their medication adherence and to develop strategies to improve hypertensive patients’ skills in dealing with the disease	N = 420 patients; A = 60.6 years	MMAS-8 (scores ranged from 0 to 8)	High adherence (score of 8)
Shimels et al. [57]	2021	Cross-sectional	To evaluate the dimension and associated factors of poor medication adherence among diabetic and hypertensive patients during the COVID-19 pandemic	N = 409 patients; A = 56.5 years	MMAS-8 (scores ranged from 0 to 8)	High adherence (score of 8)
Singh et al. [58]	2019	Cohort study	To study and identify potential risk factors for medication non-adherence	N = 8,218 patients; A = 76.6 ± 5.4 years (derivation cohort) and 76.0 ± 5.4 years (validation cohort)	PDC	Non-adherent if PDC <0.8
Sung et al. [59]	2021	Randomized controlled trial	To evaluate if a triple-component single-pill combination improved medication adherence over an equivalent two-pill combination therapy, using a Medication Event Monitoring System (MEMS)	N = 145 patients; A = 56.0 ± 15.3 years	Using MEMS data to calculate the percentage of doses taken (PDT) and the percentage of days on which the prescribed dose was taken correctly (PDTc)	High adherence if PDT ≥80% and PDTc ≥80%
Tajeu et al. [60]	2019	Cohort study	To evaluate factors in antihypertensive medication non-persistence and low adherence among adults aged <65 years	N = 379,658 patients; A = not reported	PDC	Low adherence if PDC <80%
Varleta et al. [61]	2017	Randomized controlled trial	To evaluate whether the effect of a mobile phone text messaging intervention messaging improves self-reported antihypertensive drug adherence in patients with hypertension, against a population with no text messaging	N = 314 patients; A = 60 ± 10 years	MGL (scores ranged from 0 to 4)	High adherence (score of 4)
Vázquez Machado et al. [62]	2019	Cross-sectional	To study the occurrence of depressive disorders and vital events in patients with HBP and their relationship with adherence to antihypertensive therapy	N = 222 patients; A = not reported	MMAS-8 (scores ranged from 0 to 8)	Low adherence (score of 0–5)
Vieira et al. [63]	2021	Quasi-experimental study	To study the impact of using a monthly electronic medication organizer device (Supermed) on medication adherence	N = 32 patients; A = 71.4 ± 5.6 years	MGL (scores ranged from 0 to 4)	High adherence (score of 3–4)
Wakai et al. [64]	2021	Cross-sectional	To study the importance related to the number of medications and complexity of medication regimens for medication adherence and blood pressure control in patients with hypertension	N = 1,057 patients; A = not reported	Pharmacists conducted the first interview for patients and assessed the patients’ medication adherence to brought medicines immediately upon admission	Poor adherence if the patients cannot manage medications by themselves or if they require their family caregivers and/or nurses to manage their medications
Wu et al. [65]	2018	Cohort study	To evaluate how a health-coaching intervention would affect medication adherence and blood pressure, and to study if variations in medication adherence itself would be associated with changes in blood pressure	N = 477 patients; A = 57.1 ± 12.0 years	MMAS-8 (scores ranged from 0 to 8)	High adherence (score of 6–8)

Quality of evidence and risk of bias assessment revealed that most studies included had low to moderate risk of bias (see appendices). One cross-sectional study was identified as having a high risk of bias [32] since it did not clarify the inclusion criteria, the study setting, or possible cofounders of the study or strategies to deal with them and was not clear about the statistical approach applied. One RCT was deemed to have some concerns [25] in risk of bias because the control group compared to the intervention group did not use an electronic medication tray, even though this tool was used as part of a secondary outcome measure; this slight difference between groups might have influenced the real outcomes.

Adherence to antihypertensive therapy was assessed using several tools labeled as direct and indirect. Direct measures that study the patient’s medication-related behavior using the measurement of the drug or its metabolite concentration in different body fluids such as blood or urine were only used in five studies [22,30,37,38,43]. By contrast, indirect measures were the most prevalent tools applied. Clinical assessment using questionnaires and surveys was the most prevalent tool, with the Morisky Medication Adherence Scale with 8 items (MMAS-8) being the measure more frequently used in 11 studies [24,25,30,33,45,46,52,56,57,62,65] of the 45, followed by five studies [21,32,40,61,63] that used Morisky Green Levine (MGL) scale with four items. A considerable number of other questionnaires have been used, with both objective and subjective measures, and with numerous versions to accommodate various circumstances [23,31,35,36,47,48,50,51].

One study used pharmacist-conducted interviews with patients, assessing patients’ medication adherence to the medicines brought with them, immediately on admission [64]. Measures involving secondary data analyses curated from data systems such as electronic prescription records and insurance data were commonly used, with the proportion of days covered (PDC) being assessed in nine articles [28,34,39,41,44,49,53,58,60]. Other investigations included the use of medication possession ratio (MPR) [42], medication refill adherence (MRA) [26], cumulative medication adherence (CMA) [27], the proportion of months covered (PMC) [39], percentage of doses taken (PDT), and percentage of doses taken correctly (PDTc) [59], as well as other ratios specifically calculated [29, 55]. Other adherence methods employed to study adherence included the direct count of the medication [21], the use of electronic medication packaging devices [25,54], and specialist opinion [22].

Several studies opted for a multifeatured approach, selecting two or more of the medication adherence measures previously mentioned to increase the accuracy of the information needed to correctly evaluate patients' adherence levels; e.g., one study applied four tools [MMAS-8, medication adherence rate scale (MARS), prescription refill records, and therapeutic drug monitoring (TDM)], individually and to calculate a composite score [30]. Another study regarding patients’ adherence to non-pharmacological treatment used a composed instrument that included the following parameters: weight and abdominal circumference control, practice of physical activity, and alcohol consumption [47]. Across all of the examined domains, the following associations had a constant and statistically significant association with adherence: satisfaction with communication, patient-provider relationship, digital/electronic health (eHealth) and telehealth/telemedicine services using smartphones and other technological devices (mHealth), having depression/occurrence of vital events in the patient’s life, cognitive impairment, frailty and disability, history of previous hospitalization, the type of drug, drug appearance, satisfaction with their treatment, health literacy, self-efficacy, and implicit/explicit attitudes.

Higher adherence was observed among black patients with a high level of communication with their clinicians and with higher levels of involvement in shared decision-making [26]. A better patient-provider relationship, where clinicians always explained things clearly and always listened to their patients while showing concern, led to patients being more likely to be adherent, irrespective of their ethnic group [26,33]. On the other hand, lower adherence was more common when discussions with their clinicians were less patient-centered, less psychosocially focused, and characterized by a lack of detailed discussion or clear explanation about their antihypertensive medications [54]. These three studies used a cohort study design, and when assessing the methodological quality, two of them were rated as moderate risk [26,54], and one study as low risk [33].

Both eHealth and mHealth technological solutions have shown positive effects in enhancing adherence. The SMASH app proved to increase MA and lower SBP and DBP at each subsequent evaluation during a nine-month period, managing information from medication trays and BP monitors connected to the app, and sending alerts to patients to remind them to take their medicines and monitor BP [25]. However, as previously mentioned, this RCT showed some quality concerns. The Medisafe app, used in an RCT with a low risk of bias, demonstrated an improvement in self-reported adherence, mostly in patients with previously low levels of adherence, making them moderately adherent by the end of the follow-up period, but with no changes in SBP [46]. This app involved self-reporting of medication and BP measurements, providing alerts to remind patients to take their medicines, and allowing them to designate a “Medfriend” that could access patients’ reports and work as a peer support helping them to comply with the recommended therapy.

Another RCT with a low risk of bias suggested that using mobile phone text messaging containing educative information (about diet, medication schedule, and statements regarding the importance of medication intake) seemed to improve adherence, although the decreased BP values were not statistically significant [61]. Application of telemedicine tools in a literacy-sensitive and motivational coaching program, with monthly telephone encounters with rural patients, improved medication adherence over time and demonstrated effective drops in systolic and diastolic BP, predominantly in the low adherence group at the baseline compared to the higher adherence groups in a cohort study with low risk of bias [65]. One quasi-experimental study used an electronic medication organizer equipped with an alarm clock to help adults organize and remind the daily intake of their medicines (especially for those not familiar with modern technology such as mobile phone apps) and gave healthcare professionals access to patient’s reported adherence by the device [63]. In this study, the authors observed a significant change in adherence patterns (from less to moderate adherence) and a drop in the mean SBP and DBP of 18.5 mmHg and 4.3 mmHg, respectively; however, these results must be interpreted carefully since the study seems to carry a moderate risk of bias.

Some condition-related factors are important modifiers of adherence, with non-adherence being higher among those with newly diagnosed depression, with depression symptoms, the necessity of psychopharmacological treatment, or being affected by three or more vital events (defined as adverse circumstances that occur at any stage of the patient’s life and can induce discomfort and anxiety, like the death of close family members, interpersonal conflicts, and suffering from physical illnesses) [60, 62]. A concept-wide association study utilizing text from clinical notes to identify possible risk factors for medication non-adherence found that phrases on clinical notes including “mental illness”, “anxiety/depression”, “mood”, “SSRI”, “cognition”, “confusion”, “mental status” and “memory” worked as a predictor of poor adherence [58].

Medication adherence worsens with mild cognitive impairment, even in patients without dementia [27]. It is also lower for those with a high probability of being frail and who experienced serious fall injuries following the initiation of antihypertensive medication [39,60]. Hypertensive patients with a history of previous hospitalization were unlikely to be adherent [44,49]. In contrast, one study concluded that not having a history of previous hospitalization was associated with non-adherence [39]. In the first two studies, the mean age was 47.2 and 51.7 years respectively, while it was 75 years in the third, with all three of them having large sample sizes. Based on the methodological quality assessment of the studies in the domain of comorbidities-related factors, most of the studies showed a low risk of bias, with only three being rated as moderate risk [27,44,62].

Several therapy-related factors affect adherence, the most notable being the drug class consumed, with patients who initiated angiotensin receptor blockers (ARBs) [41,49,60], calcium channel blockers (CCB) [45,49], or angiotensin-converting-enzyme inhibitors (ACE-I) [60] monotherapy more likely to be adherent, whereas those who initiated beta blockers (BB) [47,49], thiazides diuretics [38,49,60], or loop diuretics [60] tended to show low adherence. Although one study associated CCB use with poor medication adherence in a cross-sectional setting with a low risk of bias, the authors affirmed that this finding was related to the limited availability of alternative antihypertensive agents in the study setting [24]. ACE-I showed a high association with adherence, but when compared to the use of ARBs, patients under ACE-I medication had lower adherence [49]. However, the low adherence in the study previously mentioned was attributed by the authors to the population in the study (east Asians) being rarely prescribed this class since they have higher rates of adverse effects to ACE-Is (i.e., cough).

Two articles showed adherence to be highly variable between individual drugs of the same class: bendroflumethiazide and chlortalidone were associated with both the highest and lowest adherence levels respectively, and with CCB (nifedipine, felodipine, and lercanidipine) showing higher adherence compared to other classes in a cohort study with low risk of bias; however, these discrepancies are likely due to pharmacokinetic properties and/or tolerability profiles for the different drugs [43]. Another cohort study with a moderate risk of bias reported amlodipine to be more commonly associated with higher adherence when compared to Disothiazide [55]. Two studies found that the appearance of drugs had a significant role in maintaining adherence to the recommended treatment. Changes in appearance and/or name in bioequivalent drugs could negatively affect patients' adherence and lead to errors in medication-taking behavior; also, the difficulty in reading the information on the medicine packages has been associated with non-adherence [21,35]. Overall treatment satisfaction (evaluated in the following three domains: effectiveness, side effects, and convenience) was reported to be an important predictor of good adherence [24].

Health literacy played an important role in three studies presenting with low risk of bias. A statistically significant association between low adherence and health literacy levels was found, with the main domains associated being communication and decision-making skills, independently of sociodemographic characteristics [40]. Medication literacy is positively related to adherence to therapy and involves three main domains: knowledge, attitude, and behaviors (but no association was found with skill literacy) though the correlations were weak [56]. Another study demonstrated that patient’s knowledge of hypertension was associated with better adherence to both non-pharmacological (regular physical activity, weight reduction diet, and dietary salt restriction) as well as pharmacological treatment, lower and better controlled SBP and DBP, and fewer hospitalizations when compared to those with low literacy levels [50]. Poor self-efficacy in managing hypertension, which refers to the individual’s confidence in their capacity to accomplish the therapeutic recommendations, is associated with low adherence [28]. Furthermore, implicit and explicit attitudes are related to adherence, with both of them explaining a considerable variance in low pharmacy refill and self-reported adherence. Implicit attitudes seem to affect medication-taking behavior, working as a subconscious or automatic response (both in favor and against taking medications as prescribed), that may not be noticed when using self-reported adherence questionnaires [28,36].

Other determinants evaluated in the included studies neither showed a constant relationship with adherence nor any statistically significant associations: patient’s gender, age, racial/ethnic minority, educational level, marital status, employment, financial status, social support/aids, living in metropolitan areas compared to rural areas, income level of the country, healthcare provider visits or access to specialized health care use, having health insurance or access to healthcare, having other comorbidities, diagnosis of diabetes, history of cardiovascular diseases, relation with the duration of the diagnosis of HBP, smoking or drug/alcohol abuse, side effects from the pharmacological therapy, complexity of the overall medication regimen, dose regimen with antihypertensives, patient’s body mass index, beliefs regarding the disease, and acceptance of illness.

Discussion

The purpose of this study was to summarize the evidence regarding the predictors of adherence to antihypertensive therapies in studies published in the last five years, describe the limitations of the existing research, and highlight important areas to address in the future. It involved a systematic review where the predictors of adherence were identified, extracted, and categorized into five domains. The overall quality assessment of the 45 included studies revealed small concerns, with the majority of the articles exhibiting a moderate to strong methodological quality, and statistically significant association with adherence, statistically significant association with non-adherence, or non-significant association, which are summarized in Table 2.

**Table 2:** Determinants of adherence to antihypertensive therapy

Determinants of adherence		Number of studies associated with adherence	Number of studies associated with non-adherence	Number of studies with nonsignificant association
Socioeconomic	Gender	2 [31,52]	10 [21,36,38,40,43,44,47,49,51,60]	11 [22,23,27,35,40,45,48,55,56,64,65]
	Age	10 [23,31,38,43,44,47-49,55,60]	3 [21,34,64]	8 [22,24,35,45,51,52,56,65]
	Racial/ethnic minority status	1 [39]	1 [65]	3 [23,31,35]
	Education level	1 [52]		6 [23,24,31,48,51,57]
	Marital status	1 [23]	2 [28,35]	3 [23,24,35]
	Employment	2 [51,52]	1 [23]	3 [47,48,56]
	Financial status	2 [27,56]	5 [35,36,45,47,57]	3 [23,48,55]
	Social support/aid		3 [22,23,48]	1 [57]
	Living in metropolitan vs. rural areas	1 [27]	1 [51]	1 [52]
	Country income level		1 [45]	1 [45]
Healthcare system/team	Healthcare provider visits	3 [34,48,57]	2 [32,58]	1 [33]
	Specialized healthcare use	2 [37,49]	1 [44]	2 [23,24]
	Satisfaction with communication	1 [26]		
	Patient-provider relationship	2 [26,33]	1 [54]	
	Health insurance	1 [60]	1 [60]	1 [44]
	Access to healthcare	1 [27]		1 [35]
	eHealth/mHealth	5 [25,46,61,63,65]		
Condition-related	Comorbidities	3 [27,39,49]	4 [21,27,49,57]	6 [24,45,48,51,52,56]
	Having diabetes	3 [34,47,49]	3 [54,58,60]	5 [22,24,55,57,64]
	Having depression/vital events		2 [60,62]	
	History of CVD	3 [27,39,44]	2 [22,60]	3 [24,55,64]
	Cognitive impairment		2 [27,58]	
	Duration of HBP	2 [23,51]	1 [24]	4 [35,45,52,56]
	Frailty and disability		2 [39,60]	
	Drug/alcohol abuse		4 [22,24,47,57]	4 [23,24,55,64]
	Previous hospitalization	1 [39]	3 [39,44,49]	
Therapy-related	Side effects		3 [24,58,65]	2 [51,59]
	Medication regimen	3 [29,55,59]	6 [21,24,49,58,60,64]	3 [35,43,49]
	Dose regimen	8 [29,34,41,42,44,49,53,59]	5 [35,36,38,41,43]	3 [41,51,56]
	Type of drug	6 [24,41,45,49,55,60]	6 [24,38,43,47,49,60]	
	Drug appearance		2 [21,35]	
	Treatment satisfaction		1 [24]	
Patient-related	Body mass index			4 [22,45,55,64]
	Beliefs		1 [58]	1 [30]
	Health literacy	2 [50,56]	1 [40]	
	Acceptance of illness			2 [30,52]
	Self-efficacy		1 [28]	
	Implicit and explicit attitudes	1 [28]	2 [28,36]	

The role of the patient-provider relationship has been shown to promote adherence to antihypertensive therapy, with the main contributor to this relationship being the quality of the explanations provided regarding the therapy, with a patient-centered speech that addresses psychological concerns, while promoting empowerment and involvement in the decision-making process. These findings are in line with previous review articles, which illustrated the increasing importance of understanding the patient’s perspective to promote adherence and how the quality of communication (showing concern and providing information) appears to have more influence than the absolute quantity of time spent with the clinical interview [66,67]. Practitioners should investigate adherence in a blame-free environment, offer individualized solutions, and promote patients' role in making decisions about their therapy plans.

Advances in electronic and digital health technology (eHealth and mHealth) could serve as supportive tools to improve adherence. In this review, two mobile phone apps were analyzed and showed positive effects on adherence to therapy using alerts to remind patients to take their medications, using measuring tools of adherence and blood pressure control (both wireless connected to the phone or introduced through self-reported systems), and elaborating reports about individuals that allow clinicians to study their behavior towards medication [25,46]. An RCT investigating the impact of text messaging containing educative information also showed improvement in adherence [61]. Loureiro et al. conducted a systematic review on the impact of the use of mobile phone technologies and reported positive results and better effectiveness in promoting adherence, although with moderate quality and some inconsistent findings [68]. A review article found a tendency of increased medication adherence levels and better blood pressure control while using mobile phone interventions (such as text messages providing drug intake/blood pressure monitoring reminders or smartphone applications with associated alerts according to the patient’s records on their adherence behavior) [69].

A systematic review published in the European Heart Journal reported similar results [70]. Monthly telephone encounters with patients engaged in a motivational coaching program seemed to increase medication adherence over time and demonstrated effective drop changes in systolic and diastolic BP, proving that telemedicine strategies, which have become a more common practice during the COVID-19 outbreak, can be useful [65]. For older adults and/or those not familiar with modern technologies, the use of electronic multicompartment medication devices with reminder systems might improve adherence and blood pressure control [63]. However, according to a systematic review, several studies suffer from methodological limitations, such as a study included in this review [71]. All these technological interventions showed a promising non-pharmacological strategy to consider as an adjunct to enhance adherence to antihypertensive medication and to follow-up adherence trajectories, but further investigations are required.

Psychological disorders such as depression, experiencing depressive symptoms or stressful life events (vital events) were predictors of low adherence, which is in line with other literature reviews and meta-analyses [72-74]; however, this association was not always consistent as some studies reported no link to adherence [67,75]. There was also a direct association with poor self-efficacy and low medication adherence, also confirmed in previous studies [73,76]. Furthermore, depression might lead to low adherence through the mechanism of low self-efficacy, as has been previously described [77,78]. Cognitive impairment was also associated with low adherence, even in patients without dementia [27]. Similarly, Luz et al. conducted a systematic review that confirmed the association between cognitive impairment and low adherence, but the mechanisms underlying it were still not clear [79].

Measures that promote the early detection of patients with possible diagnostic of depression, cognitive impairment and individuals with higher frailty and/or presenting with disabilities, will enable physicians to adjust care to adapt to the different constraints/conditions and optimize medication management to achieve a good blood pressure control. For example, one trial found that integrated management of hypertension and depression in patients with both conditions led to better outcomes in medication adherence [80]. Other factors negatively associated with adherence to antihypertensive therapy included being frail and having a history of serious fall injuries, with similar results found in another review [81].

History of previous hospitalization was not clearly demonstrated as a determinant of adherence, since two studies showed a significant association with low adherence [44,49]; however, another study showed that not having a history of hospitalization was linked to low adherence [39]. According to an integrative review of the literature, hospitalization was a predictor of higher medication adherence [81]. One possible explanation for higher adherence in patients with a history of hospitalization might be linked to the influence of stopping and reinitiating medication, observed previously in the use of statins, but this factor should be confirmed for antihypertensive medication [82].

Persistent low antihypertensive adherence can be partly explained by explicit (conscious or deliberative) behaviors, as well as by implicit attitudes underlying a subconscious or automatic response that influences medication-taking behavior, which might be expressed through forgetfulness [28,36]. Indeed, a study included in this review found significant data where implicit and explicit attitudes accounted for an additional variation in pharmacy refill adherence, but only explicit attitudes explained the difference in self-reported questionnaires [28]. These findings were consistent with prior data observed in patients with other chronic diseases [83,84]. Therefore, implicit attitudes toward medications may explain adherence variation that could not be explained when using self-reported questionnaires, which reinforced the importance of using both objective and subjective measures when investigating patients’ medication adherence. Adults with high health literacy related to both medication and hypertension knowledge not only improved medication adherence and systolic and diastolic BP levels but also had higher engagement in non-pharmacological therapy, such as regular physical activity, weight reduction diet, and dietary salt restriction. Similar results were found in three of the reviews [73,81,85].

The drug class of the medication prescribed was shown to be linked to strengthening patient adherence. ARBs, ACE-I, and CCB classes in monotherapy had a positive correlation with adherence. Moreover, low adherence was common in patients who were prescribed BB and diuretics, and the side effects of these drugs may explain this since they had been previously considered a major factor behind poor adherence [86]. The use of BB not only affected medication adherence but also non-pharmacological treatment (i.e., physical activity), because of the dizziness due to the hypotensive effect induced by this drug. On the other hand, diuretics can cause urinary frequency and erectile dysfunction, which might not be tolerated by patients. A meta-analysis by Kronish et al. has reported higher adherence to ACE-Is and ARBs, followed by CCBs, with the lowest adherence reported in patients under diuretics and β-blockers [87]. ACE-Is are widely prescribed in Western countries, being used as a primary treatment for hypertension [88,89]. When treatment was convenient to the patient, side effects were removed, and effectiveness was achieved, patient satisfaction was attained, and that satisfaction worked as a predictor of good adherence. Barbosa et al. found that treatment satisfaction was associated with higher levels of compliance and improved persistence with the proposed therapy [90]. Other studies have also mentioned the association between adherence and satisfaction with treatment [81,91].

Changing the appearance/name of bioequivalent drugs or having difficulty in reading the information in the respective packaging seemed to negatively affect patients’ adherence, which aligns with other studies [92,93]. Patients would probably benefit from prescriptions based on the active principle of the drugs and better regulation of the packaging. The name and appearance should be the same among bioequivalent drug classes. This challenge has been previously mentioned and concrete solutions presented [94]. Even though hypertension is a very common condition and adherence to therapy has been widely studied over the past years, findings from several of the included studies demonstrated some discrepancy, with a lot of possible determinants having an inconsistent association and lack of significance, despite having been previously mentioned by other reviews as having a statistically significant association with adherence to pharmacological and non-pharmacological therapy; this brings to fore something that is well known: antihypertensive therapy adherence behavior is multifactorial, with a complex interaction associated with different factors such as like socioeconomic, healthcare system/team-related, condition-related, therapy-related, and patient-related [95,96].

Since the absence of substantial evidence does not necessarily mean the absence of effectiveness, it is wise to highlight some other predictors that may negatively influence adherence but have not been explored and come across as relevant in other reviews. These include social support and financial aid (i.e., co-payment of healthcare access/medicines), marital status, racial/ethnic minorities, previous history of cardiovascular diseases, complexity of the prescribed regimen, and acceptance of illness (since hypertension frequently presents as an asymptomatic condition). Given the multifactorial nature of medication adherence, we can easily understand that no unique intervention is usually effective in isolation and efforts should be made to apply interventions at multiple levels [97]. Additionally, these interventions should be tailored at an individual level, ensuring patient-centered care, through active involvement of patients, their families, and healthcare providers.

Study Limitations

This systematic review has a few limitations. Primarily, it included studies that used different measurement instruments and methodologies, with different cut-off points to label patients’ behavior as adherent or non-adherent. Furthermore, there was a potential risk of overestimation and recall bias since a considerable number of studies used self-reported measures. Additionally, due to the considerable heterogeneity among the included studies (especially as a consequence of having different tools to measure adherence), the diverse populations being studied (age, gender, country, socioeconomic and cultural setting) and the duration of the follow-up period among different studies, it is difficult to conduct a meta-analysis, which is considered to provide the highest level of evidence and could lead to more significant conclusions.

Another limitation was that a large proportion of the included studies used a cross-sectional design, which constrained the establishment of causal relationships and, eventually, the generalization of the findings to the targeted populations. Several other limitations in this study are those inherent to systematic reviews in general. In this review, we included only those studies found via well-known electronic databases, excluding gray literature, books, and other dissertations/studies that could be obtained manually. Reporting bias and publication bias may be present given that positive findings have a higher chance of being published, while studies with no statistically significant findings are not likely to be published in peer-reviewed journals. Hence, our findings must be interpreted within the context of our study limitations. Future research should strive to establish acceptable parameters to measure adherence and define a gold standard method, preferably involving a combination of both direct and indirect measures of adherence. Studies should also employ longitudinal designs, with longer follow-up periods that enable investigators to assess long-term adherence behaviors and trajectories over time.

## Conclusions

Adherence to both pharmacological and non-pharmacological therapy plays an important role in the control of BP in patients with hypertension, and ensuring it represents a key challenge in public health. The barriers to adherence are multiple, complex, and often interconnected between socioeconomic, patient, therapy, condition, and healthcare system levels. Hence, healthcare teams should endeavor to study patients’ non-adherence motives, using the most suitable tools to devise adherence interventions and tailor them to individual patient needs. Based on our findings, poor adherence is linked to factors such as an unsatisfactory patient-practitioner relationship and communication, a history of depression or occurrence of vital events, cognitive impairment, low health literacy and self-efficacy, presence of frailty and/or disabilities, or a history of previous hospitalization. Moreover, the antihypertensive agent chosen, with its possible side effects, and changes in medication appearance might also influence treatment satisfaction and, ultimately, explain low adherence patterns.

In this systematic review, besides traditional determinants of adherence, implicit attitudes towards medication emerged as a novel determinant that should be taken into account when determining future interventions to achieve better outcomes in patients’ therapy adherence. Furthermore, integrating new technological approaches with hybrid systems, combining eHealth and mHealth, has the potential to aid in better follow-up and improve therapy adherence, while empowering patients to deal with the condition. We strongly recommend conducting additional research with sturdier evidence and involving newer technologies.

## Appendices

**Table 3:** Joanna Briggs Institute (JBI) critical appraisal checklist for analytical cross-sectional study Q1) Were the criteria for inclusion in the sample clearly defined? Q2) Were the study subjects and the setting described in detail? Q.3) Was the exposure measured in a valid and reliable way? Q.4) Were objective and standard criteria used for measuring the condition? Q.5) Were confounding factors identified? Q.6) Were strategies to deal with confounding factors stated? Q.7) Were the outcomes measured in a valid and reliable way? Q.8) Was appropriate statistical analysis used? “✓” indicates yes; “✕” indicates no; “?” indicates unclear. Criteria: high quality (>70% of “yes” scores), moderate quality (50-69%), and low quality (<50%)

Study	Q1	Q2	Q3	Q4	Q5	Q6	Q7	Q8	% yes/risk
Avataneo et al. [22]	✓	✓	✓	✓	✓	✓	✓	✓	Low
Barbosa et al. [23]	✓	✓	✕	✕	✕	✕	✓	✓	Moderate
Berhe et al. [24]	✓	✓	✓	✓	✓	✓	✓	✓	Low
Craig et al. [28]	✓	✓	✓	✓	✓	✓	✓	✓	Low
Durand et al. [30]	✓	✓	✓	✓	✓	✓	✓	✓	Low
Fang et al. [31]	✓	✓	✕	✕	✓	✓	✕	✓	Moderate
Fernandez et al. [32]	✕	✕	✓	✓	✕	✕	✓	✕	High
Gewehr et al. [35]	✓	✓	✓	✓	✕	✕	✓	✓	Low
Gniwa et al. [36]	✓	✓	✓	✕	✕	✕	✓	✓	Moderate
Heizomi et al. [40]	✓	✓	✓	✓	✓	✓	✓	✓	Low
Ishida et al. [41]	✓	✓	✓	✓	✓	✓	✓	✓	Low
Macquart de Terline et al. [45]	✕	✕	✓	✓	✓	✕	✓	✓	Moderate
Nascimento et al. [47]	✓	✓	?	✕	✕	✕	?	✓	Moderate
Nashilongo et al. [48]	✓	✓	✓	✓	✓	✓	✓	✓	Low
Paczkowska et al. [50]	✓	✓	✕	✓	✓	✕	✓	✓	Low
Pan et al. [51]	✓	✓	✓	✓	✓	✓	✓	✓	Low
Pluta et al. [52]	✓	✓	✓	✓	✕	✕	✓	✓	Low
Shi et al. [56]	✓	✓	✓	✓	✓	✓	✓	✓	Low
Shimels et al. [57]	✓	✓	✓	✓	✕	✕	✕	✓	Moderate
Vázquez Machado et al. [62]	✕	✕	✓	✓	✕	✕	✓	✓	Moderate
Wakai et al. [64]	✓	✓	✓	✓	✓	✓	✓	✓	Low

**Table 4:** Risk Of Bias in Non-randomized Studies - of Interventions (ROBINS-I) assessment tool

Study	Confounding	Selection bias	Classification of intervention	Intended intervention	Missing data	Measures of outcome	Reported results	Overall
Arancón-Monge et al. [21]	Low	Low	Low	Low	Moderate	Moderate	Low	Moderate
Chang et al. [26]	Low	Low	Low	Low	Low	Moderate	Low	Moderate
Cho et al. [27]	Low	Moderate	Low	Low	Low	Low	Low	Moderate
Del Pinto et al. [29]	Low	Moderate	Low	Low	Low	Low	Low	Moderate
Fortuna et al. [33]	Low	Low	Low	Low	Low	Low	Low	Low
Gao et al. [34]	Low	Low	Low	Low	Low	Low	Low	Low
Gupta et al. [37]	Moderate	Moderate	Low	Low	Low	Moderate	Low	Moderate
Gupta et al. [38]	Moderate	Moderate	Moderate	Low	Low	Low	Low	Moderate
Hargrove et al. [39]	Low	Low	Low	Low	Low	Low	Low	Low
Kim et al. [42]	Low	Low	Low	Low	Low	Moderate	Low	Moderate
Kulkarni et al. [43]	Low	Low	Low	Low	Low	Low	Low	Low
Lauffenburger et al. [44]	Moderate	Moderate	Low	Low	Low	Moderate	Low	Moderate
Nishimura et al. [49]	Low	Low	Low	Low	Low	Low	Low	Low
Rea et al. [53]	Low	Low	Low	Low	Moderate	Low	Low	Moderate
Schoenthaler et al. [54]	Moderate	Moderate	Low	Low	Low	Low	Low	Moderate
Shani et al. [55]	Moderate	Low	Low	Low	Low	Low	Low	Moderate
Singh et al. [58]	Low	Low	Low	Low	Low	Low	Low	Low
Tajeu et al. [60]	Low	Low	Low	Low	Low	Low	Low	Low
Vieira et al. [63]	Moderate	Moderate	Low	Low	Low	Low	Low	Moderate
Wu et al. [65]	Low	Low	Low	Low	Low	Low	Low	Low

**Table 5:** Revised Cochrane risk-of-bias tool for randomized trials (RoB 2) assessment tool

Study	Randomization process	Deviations from the intended interventions	Missing outcome data	Measurement of the outcome	Selection of the reported result	Overall
Chandler et al. [25]	Low	Low	Low	Some concerns	Low	Some concerns
Morawski et al. [46]	Low	Low	Low	Low	Low	Low
Sung et al. [59]	Low	Low	Low	Low	Low	Low
Varleta et al. [61]	Low	Low	Low	Low	Low	Low

## References

[1] (2021). World Health Organization. Cardiovascular diseases (CVDs). https://www.who.int/news-room/fact-sheets/detail/cardiovascular-diseases-(cvds)?gad_source=1&gclid=Cj0KCQjw2uiwBhCXARIsACMvIU3ullV7CSALBVRw3gbdjNwoER9ZsMQt1uBtiY497VODH9O6maCsyEIaAoV4EALw_wcB.

[2] (2021). OECD. Health at a glance 2021: OECD indicators. https://www.oecd-ilibrary.org/social-issues-migration-health/health-at-a-glance-2021_ae3016b9-en.

[3] Raleigh V (2019). Trends in life expectancy in EU and other OECD countries: why are improvements slowing?. OECD Health Work Pap.

[4] Instituto Nacional de Estatística (2021). National Statistics Institute (site in Portuguese). Estatísticas da Saúde.

[5] (2021). Directorate-General for Health. National program for cerebrovascular diseases (site in Portuguese). Lisboa: DGS.

[6] Polonia J, Martins L, Pinto F, Nazare J (2014). Prevalence, awareness, treatment and control of hypertension and salt intake in Portugal: changes over a decade. The PHYSA study. J Hypertens.

[7] (2021). Worldwide trends in hypertension prevalence and progress in treatment and control from 1990 to 2019: a pooled analysis of 1201 population-representative studies with 104 million participants. Lancet.

[8] Fuchs FD, Whelton PK (2020). High blood pressure and cardiovascular disease. Hypertension.

[9] World Health O. Hypertension and COVID- 19: scientific brief, 17 June 2021 (2021). WHO. Hypertension and COVID-19: scientific brief. https://iris.who.int/bitstream/handle/10665/341848/WHO-2019-nCoV-Sci-Brief-Hypertension-2021.1-eng.pdf?sequence=1.

[10] Richardson S, Hirsch JS, Narasimhan M (2020). Presenting characteristics, comorbidities, and outcomes among 5700 patients hospitalized with COVID-19 in the New York City area. JAMA.

[11] Instituto Nacional de Estatística. Inquérito Nacional de Saúde 2014 (2021). National Statistics Institute. 2014 National Health Survey (site in Portuguese). Lisboa : INE.

[12] Williams B, Mancia G, Spiering W (2018). 2018 ESC/ESH guidelines for the management of arterial hypertension. Eur Heart J.

[13] Zhou B, Perel P, Mensah GA, Ezzati M (2021). Global epidemiology, health burden and effective interventions for elevated blood pressure and hypertension. Nat Rev Cardiol.

[14] Thomopoulos C, Parati G, Zanchetti A (2014). Effects of blood pressure lowering on outcome incidence in hypertension. 1. Overview, meta-analyses, and meta-regression analyses of randomized trials. J Hypertens.

[15] Martins RD, Santiago LM, Reis MT, Roque AC, Pinto M, Simões JA, Rosendo I (2019). Implications for medical activity of differences between individuals with controlled and uncontrolled hypertension. Rev Port Cardiol (Engl Ed).

[16] Burnier M (2014). Managing 'resistance': is adherence a target for treatment?. Curr Opin Nephrol Hypertens.

[17] Abegaz TM, Shehab A, Gebreyohannes EA, Bhagavathula AS, Elnour AA (2017). Nonadherence to antihypertensive drugs: a systematic review and meta-analysis. Medicine (Baltimore).

[18] De Geest S, Sabaté E (2003). Adherence to long-term therapies: evidence for action. Eur J Cardiovasc Nurs.

[19] Aronson JK (2007). Compliance, concordance, adherence. Br J Clin Pharmacol.

[20] Brown MT, Bussell JK (2011). Medication adherence: WHO cares?. Mayo Clin Proc.

[21] Arancón-Monge JM, de Castro-Cuenca A, Serrano-Vázquez Á, Campos-Díaz L, Rodríguez Barrientos R, Del Cura-González I (2020). Effects of changing the appearance of medications in safety and adherence in chronic patients over 65 years of age in primary care. CAMBIMED Study (Article in Spanish). Aten Primaria.

[22] Avataneo V, De Nicolò A, Rabbia F (2018). Therapeutic drug monitoring-guided definition of adherence profiles in resistant hypertension and identification of predictors of poor adherence. Br J Clin Pharmacol.

[23] Barbosa MEM, Bertelli EVM, Aggio CdM, Scolari GAdS, Marcon SS, Carreira L (2019). Factors associated with adherence of adults/elderly people to the treatment of arterial hypertension in primary care (Article in Portuguese). Rev Enferm UERJ.

[24] Berhe DF, Taxis K, Haaijer-Ruskamp FM, Mulugeta A, Mengistu YT, Burgerhof JG, Mol PG (2017). Impact of adverse drug events and treatment satisfaction on patient adherence with antihypertensive medication - a study in ambulatory patients. Br J Clin Pharmacol.

[25] Chandler J, Sox L, Kellam K, Feder L, Nemeth L, Treiber F (2019). Impact of a culturally tailored mHealth medication regimen self-management program upon blood pressure among hypertensive Hispanic adults. Int J Environ Res Public Health.

[26] Chang TJ, Bridges JF, Bynum M (2021). Association between patient-clinician relationships and adherence to antihypertensive medications among black adults: an observational study design. J Am Heart Assoc.

[27] Cho MH, Shin DW, Chang SA (2018). Association between cognitive impairment and poor antihypertensive medication adherence in elderly hypertensive patients without dementia. Sci Rep.

[28] Craig LS, Peacock E, Mohundro BL (2021). Implicit and explicit attitudes toward antihypertensive medications explain variation in pharmacy refill and self-reported adherence beyond traditional risk factors: potential novel mechanism underlying adherence. J Am Heart Assoc.

[29] Del Pinto R, Desideri G, Ferri C, Agabiti Rosei E (2021). Real-world antihypertensive treatment patterns, treatment adherence, and blood pressure control in the elderly: an Italian awareness-raising campaign on hypertension by senior Italia FederAnziani, the Italian Society of Hypertension and the Italian Federation of General Practitioners. High Blood Press Cardiovasc Prev.

[30] Durand H, Hayes P, Harhen B (2018). Medication adherence for resistant hypertension: assessing theoretical predictors of adherence using direct and indirect adherence measures. Br J Health Psychol.

[31] Fang J, Chang T, Wang G, Loustalot F (2020). Association between cost-related medication nonadherence and hypertension management among US adults. Am J Hypertens.

[32] Fernandez J, Mota CD, Tandron JMC, Ferrer YB, Esmores ZA (2017). Factors related with non-adherence to pharmacological treatment in hypertensive patients of the "Policlinico XX Aniversario". An educational program design. Revista de Enferm Cardiovasc.

[33] Fortuna RJ, Nagel AK, Rocco TA, Legette-Sobers S, Quigley DD (2018). Patient experience with care and its association with adherence to hypertension medications. Am J Hypertens.

[34] Gao W, Liu H, Ge C, Liu X, Jia H, Wu H, Peng X (2020). A clinical prediction model of medication adherence in hypertensive patients in a Chinese community hospital in Beijing. Am J Hypertens.

[35] Gewehr DM, Bandeira VA, Gelatti GT, Colet C, Oliveira KR (2018). Adherence to pharmacological treatment of arterial hypertension in primary health care (Article in Portuguese). Saúde Debate.

[36] Gniwa Omezzine R, Akkara A, Abdelkafi Koubaa A, Belguith Sriha A, Rdissi A, Amamou K (2019). Predictors of poor adherence to hypertension treatment. Tunis Med.

[37] Gupta P, Patel P, Štrauch B (2017). Biochemical screening for nonadherence is associated with blood pressure reduction and improvement in adherence. Hypertension.

[38] Gupta P, Patel P, Štrauch B (2017). Risk factors for nonadherence to antihypertensive treatment. Hypertension.

[39] Hargrove JL, Pate V, Casteel CH, Golightly YM, Loehr LR, Marshall SW, Stürmer T (2017). Antihypertensive adherence trajectories among older adults in the first year after initiation of therapy. Am J Hypertens.

[40] Heizomi H, Iraji Z, Vaezi R, Bhalla D, Morisky DE, Nadrian H (2020). Gender differences in the associations between health literacy and medication adherence in hypertension: a population-based survey in Heris County, Iran. Vasc Health Risk Manag.

[41] Ishida T, Oh A, Hiroi S, Shimasaki Y, Nishigaki N, Tsuchihashi T (2019). Treatment patterns and adherence to antihypertensive combination therapies in Japan using a claims database. Hypertens Res.

[42] Kim SJ, Kwon OD, Cho B, Oh SW, Lee CM, Choi HC (2019). Effects of combination drugs on antihypertensive medication adherence in a real-world setting: a Korean nationwide study. BMJ Open.

[43] Kulkarni S, Rao R, Goodman JD, Connolly K, O'Shaughnessy KM (2021). Nonadherence to antihypertensive medications amongst patients with uncontrolled hypertension: A retrospective study. Medicine (Baltimore).

[44] Lauffenburger JC, Landon JE, Fischer MA (2017). Effect of combination therapy on adherence among US patients initiating therapy for hypertension: a cohort study. J Gen Intern Med.

[45] Macquart de Terline D, Kane A, Kramoh KE (2019). Factors associated with poor adherence to medication among hypertensive patients in twelve low and middle-income Sub-Saharan countries. PLoS One.

[46] Morawski K, Ghazinouri R, Krumme A (2018). Association of a smartphone application with medication adherence and blood pressure control: the MedISAFE-BP randomized clinical trial. JAMA Intern Med.

[47] Nascimento MO, Belo RM, Araújo TL, Silva KG, Barros MD, Figueirêdo TR, Bezerra SM (2021). Factors associated to the adherence to the non-pharmachological treatment of hypertension in primary health care. Rev Bras Enferm.

[48] Nashilongo MM, Singu B, Kalemeera F (2017). Assessing adherence to antihypertensive therapy in primary health care in Namibia: findings and implications. Cardiovasc Drugs Ther.

[49] Nishimura S, Kumamaru H, Shoji S, Sawano M, Kohsaka S, Miyata H (2020). Adherence to antihypertensive medication and its predictors among non-elderly adults in Japan. Hypertens Res.

[50] Paczkowska A, Hoffmann K, Kus K (2021). Impact of patient knowledge on hypertension treatment adherence and efficacy: a single-centre study in Poland. Int J Med Sci.

[51] Pan J, Wu L, Wang H, Lei T, Hu B, Xue X, Li Q (2019). Determinants of hypertension treatment adherence among a Chinese population using the therapeutic adherence scale for hypertensive patients. Medicine (Baltimore).

[52] Pluta A, Sulikowska B, Manitius J, Posieczek Z, Marzec A, Morisky DE (2020). Acceptance of illness and compliance with therapeutic recommendations in patients with hypertension. Int J Environ Res Public Health.

[53] Rea F, Savaré L, Franchi M, Corrao G, Mancia G (2021). Adherence to treatment by initial antihypertensive mono and combination therapies. Am J Hypertens.

[54] Schoenthaler A, Knafl GJ, Fiscella K, Ogedegbe G (2017). Addressing the social needs of hypertensive patients: the role of patient-provider communication as a predictor of medication adherence. Circ Cardiovasc Qual Outcomes.

[55] Shani M, Lustman A, Vinker S (2019). Adherence to oral antihypertensive medications, are all medications equal?. J Clin Hypertens (Greenwich).

[56] Shi S, Shen Z, Duan Y, Ding S, Zhong Z (2019). Association between medication literacy and medication adherence among patients with hypertension. Front Pharmacol.

[57] Shimels T, Asrat Kassu R, Bogale G (2021). Magnitude and associated factors of poor medication adherence among diabetic and hypertensive patients visiting public health facilities in Ethiopia during the COVID-19 pandemic. PLoS One.

[58] Singh K, Choudhry NK, Krumme AA, McKay C, McElwee NE, Kimura J, Franklin JM (2019). A concept-wide association study to identify potential risk factors for nonadherence among prevalent users of antihypertensives. Pharmacoepidemiol Drug Saf.

[59] Sung J, Ahn KT, Cho BR (2021). Adherence to triple-component antihypertensive regimens is higher with single-pill than equivalent two-pill regimens: a randomized controlled trial. Clin Transl Sci.

[60] Tajeu GS, Kent ST, Huang L (2019). Antihypertensive medication nonpersistence and low adherence for adults <65 years initiating treatment in 2007-2014. Hypertension.

[61] Varleta P, Acevedo M, Akel C (2017). Mobile phone text messaging improves antihypertensive drug adherence in the community. J Clin Hypertens (Greenwich).

[62] Vázquez Machado A, Mukamutara J, Meireles Ochoa MY (2019). Depressive disorders and life events in patients with arterial hypertension and their relationship with therapeutic adherence (Article in Spanish). Multimed.

[63] Vieira LB, Reis AM, Ramos CÁ, Reis TM, Cassiani SH (2021). The use of an electronic medication organizer device with alarm to improve medication adherence of older adults with hypertension. Einstein (Sao Paulo).

[64] Wakai E, Ikemura K, Kato C, Okuda M (2021). Effect of number of medications and complexity of regimens on medication adherence and blood pressure management in hospitalized patients with hypertension. PLoS One.

[65] Wu JR, Cummings DM, Li Q, Hinderliter A, Bosworth HB, Tillman J, DeWalt D (2018). The effect of a practice-based multicomponent intervention that includes health coaching on medication adherence and blood pressure control in rural primary care. J Clin Hypertens (Greenwich).

[66] Poulter NR, Borghi C, Parati G, Pathak A, Toli D, Williams B, Schmieder RE (2020). Medication adherence in hypertension. J Hypertens.

[67] van der Laan DM, Elders PJ, Boons CC, Beckeringh JJ, Nijpels G, Hugtenburg JG (2017). Factors associated with antihypertensive medication non-adherence: a systematic review. J Hum Hypertens.

[68] Loureiro RM, de Azevedo DA (2021). Therapeutic adherence in hypertensive patients: review of the role of new platforms via cell phone or smartphone (Article in Portuguese). Portuguese J Family Med and Gen Pract.

[69] Andre N, Wibawanti R, Siswanto BB (2019). Mobile phone-based intervention in hypertension management. Int J Hypertens.

[70] Gandapur Y, Kianoush S, Kelli HM (2016). The role of mHealth for improving medication adherence in patients with cardiovascular disease: a systematic review. Eur Heart J Qual Care Clin Outcomes.

[71] Paterson M, Kinnear M, Bond C, McKinstry B (2017). A systematic review of electronic multi-compartment medication devices with reminder systems for improving adherence to self-administered medications. Int J Pharm Pract.

[72] Grenard JL, Munjas BA, Adams JL, Suttorp M, Maglione M, McGlynn EA, Gellad WF (2011). Depression and medication adherence in the treatment of chronic diseases in the United States: a meta-analysis. J Gen Intern Med.

[73] Peacock E, Krousel-Wood M (2017). Adherence to antihypertensive therapy. Med Clin North Am.

[74] Holt EW, Muntner P, Joyce C, Morisky DE, Webber LS, Krousel-Wood M (2012). Life events, coping, and antihypertensive medication adherence among older adults: the cohort study of medication adherence among older adults. Am J Epidemiol.

[75] Bochkareva EV, Butina EK, Kim IV, Kontsevaya AV, Drapkina OM, Leon D, McKee M (2019). Adherence to antihypertensive medication in Russia: a scoping review of studies on levels, determinants and intervention strategies published between 2000 and 2017. Arch Public Health.

[76] Yue Z, Li C, Weilin Q, Bin W (2015). Application of the health belief model to improve the understanding of antihypertensive medication adherence among Chinese patients. Patient Educ Couns.

[77] Schoenthaler A, Ogedegbe G, Allegrante JP (2009). Self-efficacy mediates the relationship between depressive symptoms and medication adherence among hypertensive African Americans. Health Educ Behav.

[78] Chao J, Nau DP, Aikens JE, Taylor SD (2005). The mediating role of health beliefs in the relationship between depressive symptoms and medication adherence in persons with diabetes. Res Social Adm Pharm.

[79] Alencar Luz AL, Griep RH, Landim MB, Alencar DD, Macedo JB (2021). Adherence to antihypertensive treatment in elderly people with cognitive impairment: systematic review (Article in Portuguese). Revista Cogitare Enferm.

[80] Bogner HR, de Vries HF (2008). Integration of depression and hypertension treatment: a pilot, randomized controlled trial. Ann Fam Med.

[81] Oori MJ, Mohammadi F, Norouzi K, Fallahi-Khoshknab M, Ebadi A (2019). Conceptual model of medication adherence in older adults with high blood pressure-an integrative review of the literature. Curr Hypertens Rev.

[82] Brookhart MA, Patrick AR, Schneeweiss S (2007). Physician follow-up and provider continuity are associated with long-term medication adherence: a study of the dynamics of statin use. Arch Intern Med.

[83] Linn AJ, Vandeberg L, Wennekers AM, Vervloet M, van Dijk L, van den Bemt BJ (2016). Disentangling rheumatoid arthritis patients' implicit and explicit attitudes toward methotrexate. Front Pharmacol.

[84] Rüsch N, Todd AR, Bodenhausen GV, Weiden PJ, Corrigan PW (2009). Implicit versus explicit attitudes toward psychiatric medication: implications for insight and treatment adherence. Schizophr Res.

[85] Egan BM, Zhao Y, Axon RN (2010). US trends in prevalence, awareness, treatment, and control of hypertension, 1988-2008. JAMA.

[86] Osterberg L, Blaschke T (2005). Adherence to medication. N Engl J Med.

[87] Kronish IM, Woodward M, Sergie Z, Ogedegbe G, Falzon L, Mann DM (2011). Meta-analysis: impact of drug class on adherence to antihypertensives. Circulation.

[88] Schulz M, Krueger K, Schuessel K, Friedland K, Laufs U, Mueller WE, Ude M (2016). Medication adherence and persistence according to different antihypertensive drug classes: a retrospective cohort study of 255,500 patients. Int J Cardiol.

[89] Kurdi AI, Chen LC, Elliott RA (2017). Exploring factors associated with patients' adherence to antihypertensive drugs among people with primary hypertension in the United Kingdom. J Hypertens.

[90] Barbosa CD, Balp MM, Kulich K, Germain N, Rofail D (2012). A literature review to explore the link between treatment satisfaction and adherence, compliance, and persistence. Patient Prefer Adherence.

[91] Shahin W, Kennedy GA, Stupans I (2021). The association between social support and medication adherence in patients with hypertension: a systematic review. Pharm Pract (Granada).

[92] Håkonsen H, Eilertsen M, Borge H, Toverud EL (2009). Generic substitution: additional challenge for adherence in hypertensive patients?. Curr Med Res Opin.

[93] Greene JA, Kesselheim AS (2021). Why do the same drugs look different? Pills, trade dress, and public health. N Engl J Med.

[94] Ciociano N, Bagnasco L (2014). Look alike/sound alike drugs: a literature review on causes and solutions. Int J Clin Pharm.

[95] Krousel-Wood M, Joyce C, Holt E (2011). Predictors of decline in medication adherence: results from the cohort study of medication adherence among older adults. Hypertension.

[96] Krousel-Wood M, Thomas S, Muntner P, Morisky D (2004). Medication adherence: a key factor in achieving blood pressure control and good clinical outcomes in hypertensive patients. Curr Opin Cardiol.

[97] La Caze A, Gujral G, Cottrell WN (2014). How do we better translate adherence research into improvements in patient care?. Int J Clin Pharm.

